# Hydroxylation index of omeprazole in relation to CYP2C19 polymorphism and sex in a healthy Iranian population

**DOI:** 10.1186/s40199-014-0081-6

**Published:** 2014-12-11

**Authors:** Maryam Payan, Mohammad Reza Rouini, Nader Tajik, Mohammad Hossein Ghahremani, Reza Tahvilian

**Affiliations:** Biopharmaceutics and Pharmacokinetics Division, Department of Pharmaceutics, School of Pharmacy, Tehran University of Medical sciences, Tehran, Iran; Cellular and Molecular Research Center (CMRC), Iran University of Medical Sciences, Tehran, Iran; Department of Pharmacology and Toxicology, School of Pharmacy, Tehran University of Medical sciences, Tehran, Iran; Department of pharmaceutics, School of Pharmacy, Kermanshah University of Medical Sciences, Kermanshah, Iran

**Keywords:** CYP2C19, Enzyme activity, Genotype, Omeprazole, Phenotype

## Abstract

**Background:**

Polymorphism of *CYP2C19* gene is one of the important factors in pharmacokinetics of CYP2C19 substrates. Omeprazole is a proton pump inhibitor which is mainly metabolized by cytochrome P450 2C19 (*CYP2C19*). The aim of present study was to assess omeprazole hydroxylation index as a measure of CYP2C19 activity considering new variant allele (CYP2C19*17) in Iranian population and also to see if this activity is sex dependent.

**Methods:**

One hundred and eighty healthy unrelated Iranian individuals attended in this study. Blood samples for genotyping and phenotyping were collected 3 hours after administration of 20 mg omeprazole orally. Genotyping of *2C19* variant alleles *2, *3 and *17 was performed by using polymerase chain reaction-restriction fragment length polymorphism (PCR-RFLP) and semi-nested PCR methods. Plasma concentrations of omeprazole and hydroxyomeprazole were determined by high performance liquid chromatography (HPLC) technique and hydxroxylation index (HI) (omeprazole/ hydroxyomeprazole) was calculated.

**Results:**

The CYP2C19*17 was the most common variant allele in the studied population (21.6%). Genotype frequencies of CYP2C19*17*17, *1*17, and *2*17 were 5.5%, 28.8% and 3.3% respectively. The lowest and the highest median omeprazole HI was observed in *17*17 and *2*2 genotypes respectively (0.36 vs. 13.09). The median HI of omeprazole in subjects homozygous for CYP2C19*1 was 2.16-fold higher than individuals homozygous for CYP2C19*17 (P < 0.001) and the median HI of CYP2C19*1*17 genotype was 1.98-fold higher than CYP2C19 *17*17 subjects (P < 0.001). However, subjects with CYP2C19*2*17 (median HI: 1.74) and CYP2C19*1*2 (median HI: 1.98) genotypes and also CYP2C19*1*17 (median HI: 0.71) and CYP2C19*1*1 (mean HI: 0.78) did not show any significantly different enzyme activity. In addition, no statistically significant difference was found between women and men in distribution of *CYP2C19* genotypes. Furthermore, the hydroxylation index of Omeprazole was not different between women and men in the studied population.

**Conclusion:**

Our data point out the importance of CYP2C19*2 and CYP2C19*17 variant alleles in metabolism of omeprazole and therefore CYP2C19 activity. Regarding the high frequency of CYP2C19*17 in Iranian population, the importance of this new variant allele in metabolism of CYP2C19 substrates shall be considered.

## Introduction

Cytochrome P450 includes a wide variety of phase I metabolizing enzymes which are involved in metabolism of drugs and endogenic substances [1,2]. CYP2C19 is one of the members of cytochrome iso enzyme superfamily which contributes in metabolism of important drugs such as proton pump inhibitors (PPI) [3] psychotic drugs like venlafaxine [4] and citalopram [5,6], voriconazol [7], and clopidogrel [8,9].

CYP2C19 is represented by a gene located on chromosome 10 [10]. Genetic polymorphism of CYP2C19 is one of the major reasons of inter-individual variability in response to CYP2C19 substrate [11-13]. The main CYP2C19 polymorphisms that are associated with difference in therapeutic response are attributed to CYP2C19*2, CYP2C19*3 and CYP2C19*17 [14,15].

A point mutation in exon 5 (*681 G > A*, designated *2) causes a cryptic splice defect (CYP2C19*2) and a single nucleotide polymorphism (SNP) in exon 4 (*636 G > A* designated *3) creates a stop codon. Both mutations predominantly result in decreased CYP2C19 activity [9,16]. A recently discovered SNP in 5′ –flanking region *(−806 C > T* and −*3402 C > T*) leads to increased CYP2C19 activity and therefore produces ultra rapid metabolizer phenotype [17,18].

The CYP2C19*2*2 and *3*3 genotypes are more prevalent in oriental and Asian populations than in Caucasian (12-23% vs 3-5%). In contrast the CYP2C19*17*17 is more frequent in Caucasian than in Asian populations (18-26% vs 0.4-1.4%) [19,20].

Omeprazole is a proton pump inhibitor that is administered in treatment of gastric acid related disease [21]. Polymorphism of CYP2C19 can affect pharmacokinetic and therefore efficacy of proton pump inhibitors [21,22]. Additionally non genetic factors like age, liver disease and combination therapy can result in resistance to *Helicobacter Pylori* eradication treatment [23,24].

Several studies have used hydroxylation index of omeprazole as an indicator of CYP2C19 activity however this enzyme activity, was mainly measured in relation to *2 and *3 variant alleles and not the new variant allele (*17) [16,25-28]. Although Sim *et al*. studied the effect of CYP2C19*17 variant allele on enzyme activity, they only reported this activity in extensive metabolizers (*17*17, *1*17 and *1*1) and they did not determine CYP2C19 activity in CYP2C19*2*17 carriers [17]. CYP2C19*2 leads to decreased enzyme activity and CYP2C19*17 causes increased enzyme activity [16,17] but the impact of combined alleles (CYP2C19*2*17) on CYP2C19 activity has not been reported comprehensively and it is unknown that the effect of which allele is more predominant in CYP2C19*2*17 carriers.

Furthermore there are some controversies in publications about impact of sex on CYP2C19 activity [29,30]. To our best knowledge, currently there is no published data regarding CYP2C19 activity in relation to new variant allele in Iranian population. Thus, the objects of this study were to assess effect of CYP2C19*17 on enzyme activity and also to see if there is any sex-dependent difference in CYP2C19 activity and finally to investigate genotype-phenotype relationship of CYP2C19 considering new variant allele (CYP2C19*17) in Iranian population.

## Material and methods

### Study subjects

The study protocol was approved by ethics committee of Tehran University of Medical Sciences (ethical no. 11208). Generally one hundred and eighty (60 women and 120 men) unrelated healthy Iranian volunteers with the mean age of between 20–55 years and average body weight of 45–89 kg took part in this study. All participants signed written informed consent of this project. The study was completed by contribution of faculties of pharmacy of Tehran, Yazd, Kermanshah and Kerman University of Medical Sciences. The participants were students or stuffs of pharmacy schools, with no history of any illness or medicine consumption. No smoking and consumption of medicine that would affect CYP2C19 activity was permitted for one week before and during the study.

### CYP2C19 phenotyping

After an overnight fast for at least 8 hours, volunteers took 20 mg omeprazole capsule (Abidi pharmaceuticals) with 250 milliliter tap water. Ten ml venous blood sample was collected from each subject 3 hours after administration of omeprazole and transferred into tubes containing 10 μl of 10% EDTA. Five ml of blood samples were centrifuged for 5 min at 4000 rpm and the plasma was separated and transferred to Eppendorf tube and stored at −80°C up to the day of analysis. The other 5 ml blood samples were stored directly in −80°C for genotyping analysis.

### Analytical procedure

Omeprazole powder was purchased from TMAD (Iran). 5-hydroxyomeprazole was a kind donation by AstraZeneca (Sweden). The concentration of omeprazole and 5-hydroxyomeprazole was analyzed by HPLC method as described by Rezk *et al.* with a few modifications [31]. Briefly 500 μl plasma was extracted by liquid-liquid extraction using 1500 μl ethyl acetate. After orbital mixing for 10 min and centrifuging at 4000 × g for 10 min, the upper organic layer was separated and transferred to glass tube and then evaporated to dryness under gentle stream of air. Finally the residue was dissolved in 250 μl mobile phase and 100 μl of this sample was injected to HPLC system. The mobile phase was a combination of dibasic sodium phosphate buffer (0.025 mol/lit, pH 6): acetonitrile: methanol (73: 18: 8 V/V/V). The HPLC apparatus consisted of a low pressure HPLC pump, UV detector (λ = 302 nm) all from Knauer (Berlin, Germany). The chromatographic separation was performed by using Chromolit™ Performance RP-18e 100 mm × 4.6 mm, 5 μm particle size. Flow rate was adjusted to 1 ml/min. The limits of quantification were about 15 μg/ml for both compounds. Intraday and between day precisions were < 5% for both omerpazole and 5-hydroxyomeprazole.

### *CYP2C19* genotyping

The DNA was extracted from blood leucocytes by standard salting out method as explained by Miller *et al*. [32]. The extracted DNA was dissolved in sterile distilled water and stored at 4 °C until the day of analysis. Amplification of CYP2C19*2 and *3 allele was implemented using polymerase chain reaction-restriction fragment length polymorphism (PCR-RFLP) as described by De Morias [33]. The PCR product of each reaction was digested by specific endonuclease (all from New England Biolabs GmbH, Frankfurt, Germany); the 169 bp CYP2C19*2 product was digested by SmaI to 40 and 129 bp fragments. The 329 bp PCR product of CYP2C19*3 was digested by BamHI to 233 and 96 bp pieces. Genotyping of CYP2C19*17 -3402 C > T and −*806 C > T* polymorphisms was done by PCR-RFLP and nested-PCR assays as defined by Sim *et al*. [17]. For CYP2C19*17 -3402 C > T the PCR product (504 bp) was digested by MnlI and resulted in 224 and 280 bp fragments. But the PCR product of CYP2C19*17 -806 C > T (200 bp) was separated directly on 2.5% agarose gel without any digestion. In all PCR-RFLP assays mutation caused abolishment of restriction site and thus PCR product was not digested.

### Statistical analysis

The allele frequencies differences between population were estimated using two-tailed Fisher’s exact test. The 95% confidence intervals (CI) were calculated using Confidence Interval Analysis software. The relation of sex and genotype was assessed by two tailed Fisher’s exact test. The observed and expected frequencies were calculated by using Hardy-Weinberg equation. The two-tailed Fisher’s exact test was used to evaluate deviation of genotype frequencies in the studied population from Hardy-Weinberg equilibrium. The enzyme activity was compared by using omeprazole hydroxylation index. The hydroxylation index (HI) of omeprazole 3 hours after administration of omeprazole was calculated by dividing omeprazole to 5-hydroxyomeprazol plasma concentration. The mean HI in different genotypes were compared by Mann–Whitney two tailed test. The impact of sex on HI of omeprazole was also evaluated using Mann–Whitney two tailed test. The inter-individual variability in metabolism of omeprazole was represented by probit plot. For drawing probit plot, the log of HI was calculated, the antimode value was determined using Microsoft office excel 2010. The normality of HI distribution was analyzed by frequency distribution histogram and also by Kolmogorov- Smirnov test. All statistical analyses were performed by Sigma Plot version 12.0 and Graph Pad Prism version 5 softwares and P < 0.05 was considered as statistically significant difference.

## Results

The genotype and allele frequencies of CYP2C19 are reported in Table 1. According to the data presented in Table 1, CYP2C19 *17*17, *1*17 and *1*1 were detected in 10 (5.5%), 52 (28.9%) and 75 (41.7%) subjects respectively. The CYP2C19 *2*17 and *1*2 were identified in 6 (3.3%) and 33 (18.3%) individuals and finally the CYP2C19*2*2 was recognized in 4 (2.2%) of volunteers. CYP2C19*17 was the most common variant allele in Iranian population.

**Table 1:** **Genotype and allele frequencies of CYP2C19 in 180 healthy Iranian volunteers**

**CYP2C19 Genotype**	**Number of subjects**		**Frequency (%)**	**95% CI**
	**Men (120)**	**Women (60)**		
*17*17	5	5	5.5	2.7 - 10.0
*1*17	37	15	28.8	22.4 - 36.1
*1*1	53	22	41.7	34.4 - 49.2
*1*2	20	13	18.3	13.0 - 24.7
*2*17	4	2	3.3	1.23 - 7.1
*2*2	1	3	2.2	0.6 - 5.5
**CYP2C19 Alleles**	**No. of alleles**		**Frequency (%)**	**95% CI**
CYP2C19*17	78		21.6	17.5 - 26.3
CYP2C19*1	235		65.3	60.1 - 70.2
CYP2C19*2	47		13.1	9.7 - 16.9
CYP2C19*3	0		0	0

*CI**:** Confidence Interval. (The 95% confidence intervals (CI) were calculated using Confidence Interval Analysis software).

The hydroxylation index of omeprazole as mean ± SD, median and 95% confidence interval is reported in Table 2. Subjects with CYP2C19 *17*17 genotype had a very high metabolic capacity with median hydroxylation index of 0.36 and were classified as Ultra-Rapid Metabolizers (URM). The median hydroxylation index of omeprazole in subjects homozygous for CYP2C19*1 was 2.17 fold higher than individual homozygous for CYP2C19*17 (P < 0.001) and the median hydroxylation index of CYP2C19*1*17 genotype was 1.97 fold higher than CYP2C19*17*17 subjects (P < 0.001). There was not a significant difference between HI of omeprazole in CYP2C19*1*17 and *1*1 carriers (P > 0.05) and these two groups were stratified as extensive metabolizers (EM).

**Table 2:** **Hydroxylation index of omeprazole (omeprazole/hydroxyomeprazole) in relation to CYP2C19 genotype in 180 healthy Iranian subjects**

	*17*17	*1*17	*1*1	*2*17	*1*2	*2*2
Mean (SD)	0.35(0.06)	0.75(0.28)	0.85(0.30)	2.02(0.84)	2.27(1.04)	13.59(3.13)
Median	0.36^a^	0.71	0.78	1.74^b^	1.98^b^	13.03^a^
95% CI	0.31 - 0.39	0.68 -0.83	0.79 – 0.92	1.33 – 2.72	1.92 – 2.63	10.51– 16.67

^a^Represent statistically significant difference with other 5 genotypes.

^b^Represent statistically significant difference with *17*17, *1*17, *1*1 and *2*2.

CI: Confidence Interval.

The median HI of omeprazole was 1.74 in CYP2C19*2*17 and 1.98 in CYP2C19*1*2 carriers respectively. The difference in HI of omeprazole in CYP2C19*1*2 carriers were statistically significant with other CYP2C19 genotypes (P < 0.05) except for CYP2C19*2*17 genotype (p > 0.05). Individuals in these two groups had intermediate metabolic capacity and were designated as Intermediate-Metabolizers (IM). Homozygous carrier of CYP2C19*2 had a very low metabolic capacity with the median hydroxylation index of 13.03 and they were classified as poor metabolizers (PM). There was a significant difference between HI index of homozygous carriers of CYP2C19*2 with the other five genotypes (p < 0.001).

**Figure 1:**
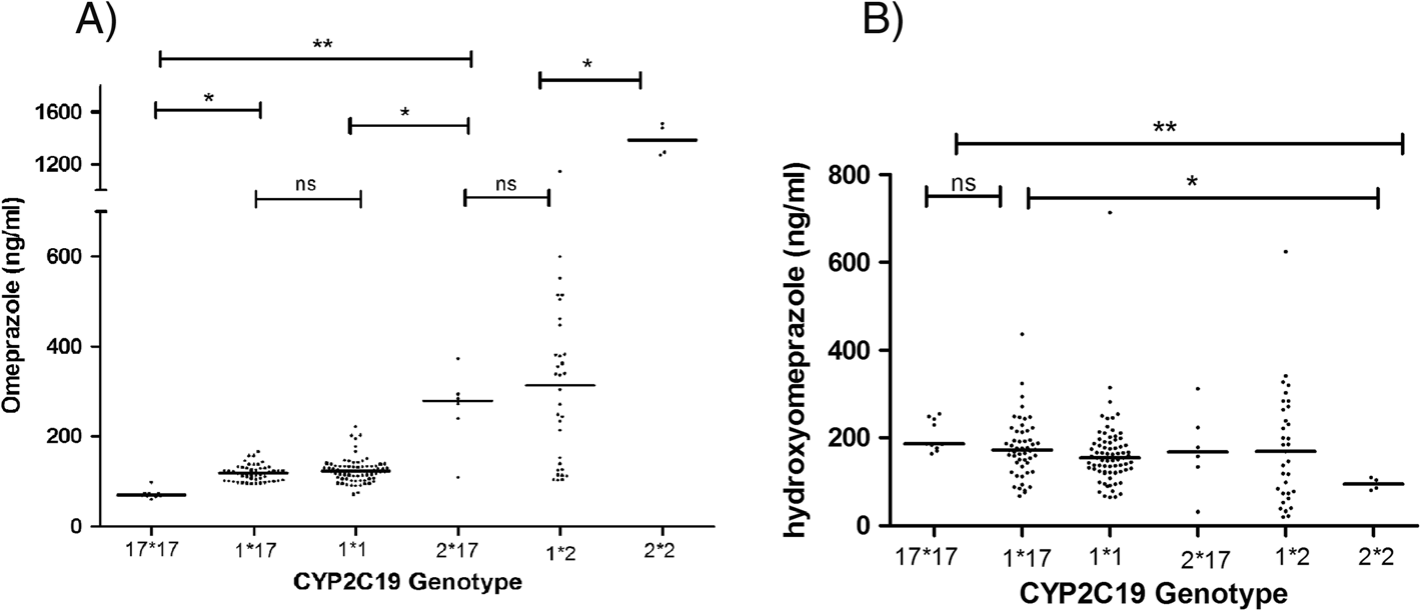
**Plasma concentrations of Omeprazole (A) and hydroxyomerpazole (B) in different genotypes 3 hours after administration of Omeprazole orally.** ns: not significant, * p < 0.05, ** p < 0.001.

The plasma concentration of omeprazole and hydroxyomeprazole is illustrated in Figure 1. According to this figure there is a significant difference between omeprazole plasma concentration in individuals with CYP2C19*17*17 genotype with all other groups (P < 0.01), but the omeprazole plasma concentration was neither different between 1*17 and 1*1(EM) (P > 0.5) nor between 2*17 and 1*2 (IM) genotypes. However, omeprazole plasma concentration was significantly different between EM (1*17 and 1*1) and IM (2*17 and 1*2). The plasma concentration of hydroxyomeprazole was significantly different between *2*2 and all other 5 genotypes while the plasma concentration of hydroxyomeprazole was not significantly different between other 5 genotypes (17*17, 1*17, *1*1, 2*17, 1*2).

**Figure 2:**
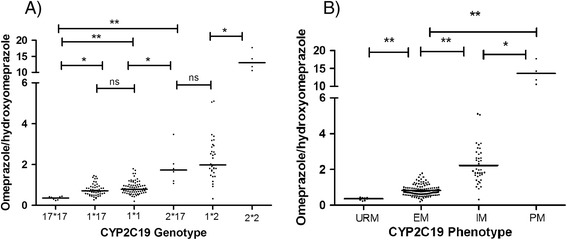
**The hydroxylation index of Omeprazole in different genotypes (A) and in predicted phenotype groups (B) 3 hours after administration of Omeprazole orally.** ns: not significant, * p < 0.05, ** p < 0.001.

Figure 2 indicates the hydroxylation index of omeprazole in 6 genotypes and also in predicted phenotype groups. As it is observed there is no significant difference between hydroxylation index of omeprazole in 1*17 and 1*1 groups or between 2*17 and 1*2 groups. While the difference between 17*17 or 2*2 with all other genotype groups were statistically significant.

The summary of omeprazole, hydroxyomeprazole plasma concentration and omeprazole HI in the total population, women and men is reported in Table 3. Omeprazole plasma concentration was significantly higher in 2*2 genotype than other genotype groups. Additionally there was a significant difference in omeprazole plasma concentration between different groups (P < 0.001) except for CYP2C19*1*17 and *1*1 (P > 0.05), CYP2C19*1*2 and CYP2C19*2*17 (P > 0.05). Mean omeprazole plasma concentration was 19.0 fold higher in CYP2C19*2*2 than CYP2C19*17*17 and 11 fold higher than CYP2C19*1*1 (P < 0.001), however hydroxyomeprazole concentration was not statistically different among genotype groups (P > 0.05) except for CYP2C19*2*2. Moreover, omeprazole and hydroxyomeprazole plasma concentrations as well as omeprazole HI were not statistically different among women and men in the studied population (P > 0.05).

**Table 3:** **Plasma concentration of omeprazole (OMP) and hydroxyomeprazole (OH-OMP) and hydroxylation index (HI) of omeprazole in relation to genotype in 60 women and 120 men 3 hour after administration of single oral dose of 20 mg omeprazole**

**Genotype**	**No of subjects**	**Mean OMP concentration (ng/ml) ± SD**	**Mean OH-OMP concentration (ng/ml) ± SD**	**Mean HI ± SD**
17*17	Total (10)	71.01 ± 10.28	203.31 ± 34.93	0.35 ± 0.06
	Women (5)	68.32 ± 5.73	204.92 ± 41.35	0.34 ± 0.08
	Men (5)	73.71 ± 13.67	201.69 ± 32.08	0.36 ± 0.04
1*17	Total (52)	118.28 ± 10.19	177.05 ± 37.53	0.75 ± 0.28
	Women (15)	120.22 ± 20.12	174.51 ± 28.01	0.72 ± 0.18
	Men (37)	117.49 ± 16.10	178.07 ± 44.57	0.77 ± 0.32
1*1	Total (75)	124.75 ± 27.18	163.74 ± 32.25	0.85 ± 0.30
	Women (22)	128.06 ± 39.90	194.40 ± 60.84	0.78 ± 0.27
	Men (53)	123.38 ± 20.83	151.01 ± 26.38	0.89 ± 0.30
2*17	Total (6)	289.01 ± 97.45	172.79 ± 63.58	2.02 ± 0.54
	Women (4)	324.25 ± 39.90	178.72 ± 33.59	1.97 ± 0.62
	Men (2)	271.39 ± 118.56	169.82 ± 84.95	2.05 ± 0.67
1*2	Total (33)	319.45 ± 150.34	177.79 ± 38.39	2.27 ± 1.04
	Women (13)	364.92 ± 265.16	194.66 ± 52.56	2.24 ± 0.79
	Men (20)	289.89 ± 167.55	165.53 ± 23.55	2.29 ± 1.19
2*2	Total (4)	1388.99 ± 123.41	104.67 ± 10.55	13.59 ± 3.13
	Women (3)	1359.84 ± 133.23	105.03 ± 13.80	13.37 ± 3.80
	Men (1)	1476.4	103.58	14.25

HI = (omerpazole concentration/hydroxyomeprazole concentration).

**Figure 3:**
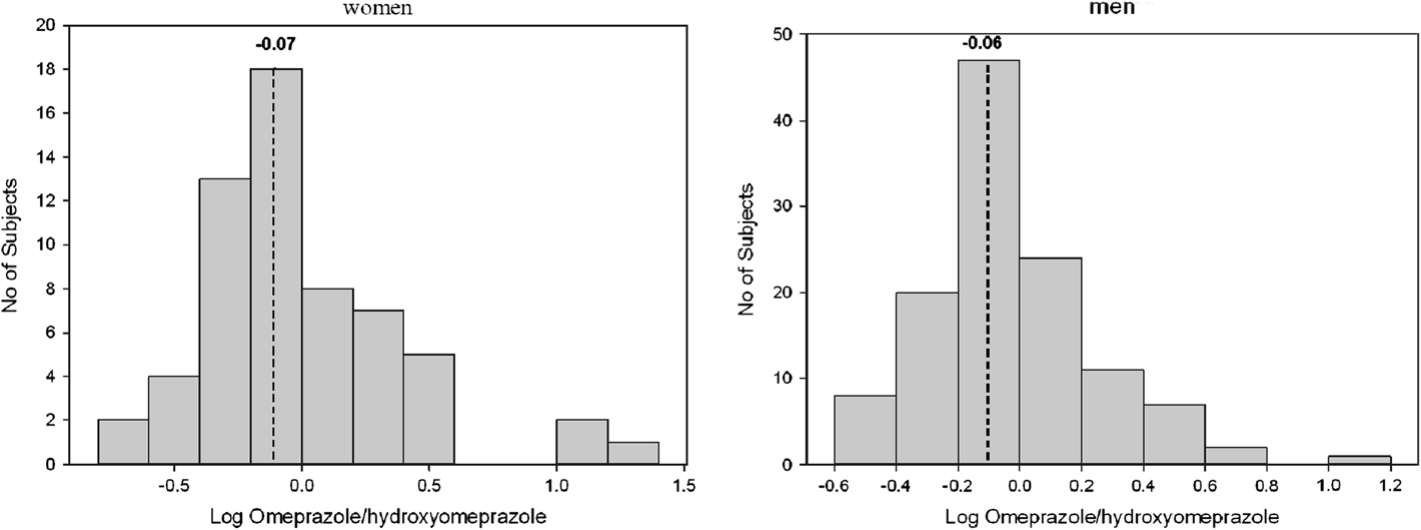
**The effect of sex on hydroxylation index of omeprazole in 60 women and 120 men.** The median hydroxylation index is indicated by dashed line.

The effect of sex on hydroxylation index of omeprazole is illustrated in Figure 3. According to this figure there is not any significant difference between median hydroxylation index in women (0.84) and men (0.86) (p > 0.05).

**Figure 4:**
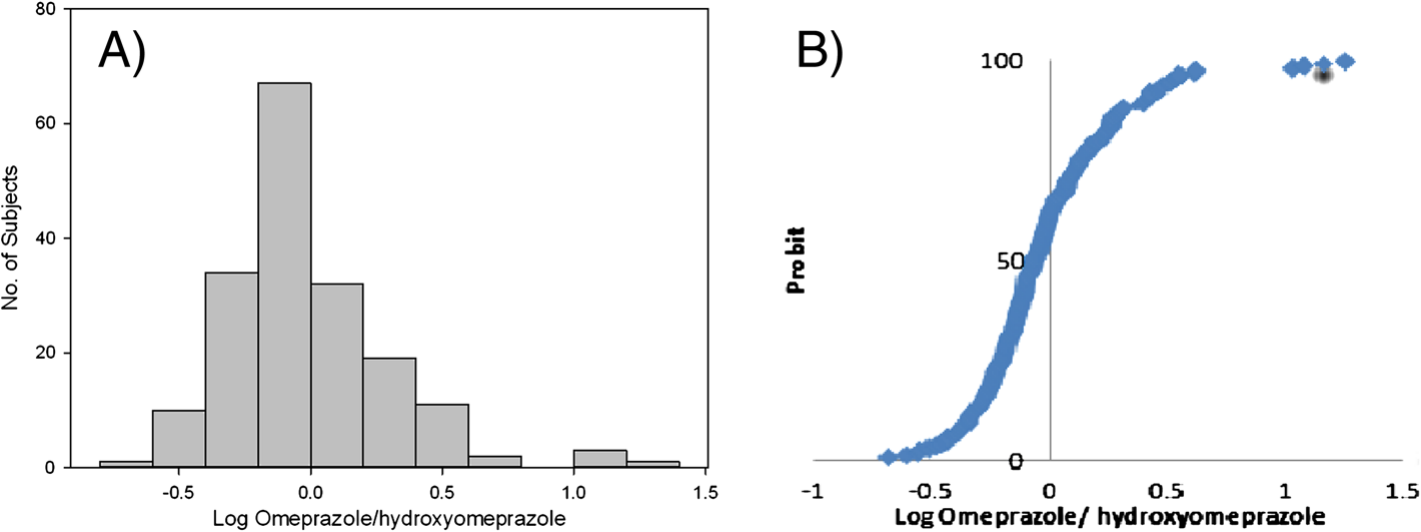
**A) Frequency histogram distribution and B) Probit plot of log omeprazole hydroxylation index in 180 healthy Iranian volunteers.** Subjects with log HI > 1.0 were phenotyped as poor metabolizers.

The frequency distribution histogram of omeprazole hydroxylation index in 180 healthy Iranian volunteers is indicated in Figure 4. The graph shows a bimodal distribution with the antimode of around 0.8. Kolmogorov-Smirnov test showed that the omeprazole hydroxylation index was not normally distributed in the studied population (K-S Dist. = 0.296 p < 0.001). The bimodal distribution was also confirmed by probit plot.

The correlation of CYP2C19 genotype and phenotype was tested using Spearman rank correlation, and the results showed a well correlation between CYP2C19 genotype and phenotype (r_s_ = 0.64, P < 0.0001).

## Discussion

Inter-individual variability in drug response always has been one of the main concerns in drug discovery and development. The important factors resulting in such variation include genetic, nongenetic and physiologic agents like change in protein structure, combination therapy, alcohol, smoking, sex, age and disease condition [34].

CYP2C19*17 is a new variant allele which is associated with increased gene transcription and therefore higher enzyme activity [17]; which may lead to several clinical consequences including the lower susceptibility to breast cancer risk [35], higher risk of peptic ulcer disease [36], greater response to clopidogrel treatment and more risk of bleeding [37] in addition to a better treatment with tamoxifen [38].

In this study, omeprazole HI after 3 hours administration of omeprazole was used as indicator of CYP2C19 activity. The HI in CYP2C19*17*17 was significantly different with CYP2C19*1*17 and CYP2C19*1*1 genotypes and people in this group had very high metabolic activity, which is in agreement with what was reported by Sim *et al*. They found that median HI of omeprazole in homozygous carriers of CYP2C19*1 is 2 fold higher than homozygous carriers of CYP2C19*17 and 1.2 fold higher than CYP2C19*1*17 [17]. Ramsjö *et al*. has also reported that mean HI of omeprazole in CYP2C19*1*1 was 3.2 fold higher than CYP2C19*17*17 and 1.1 fold higher than CYP2C19*1*17 [27].

CYP2C19*2 allele is associated with decreased enzyme activity and CYP2C19*17 variant allele is connected with increased enzyme activity. In Most of the genotype phenotype studies of CYP2C19*17 variant allele, only HI of omeprazole in CYP2C19*17*17, CYP2C19*1*17 and CYP2C19*1*1 genotypes has been reported [17,27]. However, the capacity of CYP2C19 enzyme activity in people carrying both defective mutant alleles of *2 and *17 (CYP2C19*2*17) was still unclear. Although Ragia *et al*. in the study for evaluation of distribution of CYP2C19*17 genetic polymorphism in Greece people defined CYP2C19*2*17 carriers as EM and people with CYP2C19*1*2 as IM, they only predicted phenotype based on genotype and CYP2C19 activity was not determined by using a probe drug [39]. Sugimito *et al*. did not see any difference between metabolic capacities of CYP2C19*1*1, *1*17, 2*17 and 1*2 for metabolism of omeprazole and stratified these individuals as EM [20]. The omeprazole HI in subjects with CYP2C19*2*17 genotype in this study was not significantly different from CYP2C19*1*2 genotype (P = 0.33) so we designated them as IM. It seems that in heterozygous carriers of CYP2C19*2 and *17 allele, the effect of *2 allele is more predominant than *17 allele and it can suppress induced enzyme activity by *17 allele. This observation is in agreement with classification of CYP2C19*2*17 as IM by Gurbel *et al*. based on the study for genotype phenotype analysis of 2C19 in stented patient [40]. In contrast to these findings, in a study for evaluation of CYP2C19 enzyme activity in Turkish children using lansoprazole as a probe drug, individuals with CYP2C19*2*17 had similar enzyme activity to CYP2C19*1*17 and CYP2C19*1*1; and this activity was significantly different from CYP2C19*1*2 [41]. Involvement of individual with different age groups could possibly explain such different observations. Our study was conducted in adult individuals with the average age of 32 years but Gumus implemented the study in children with mean age of 10.2 years. The lower frequency of CYP2C19*2*17 in our studied subjects in comparison to Turkish individuals (6 vs 16) can be considered as another justification.

In the present study effect of CYP2C19 genetic polymorphism and sex on metabolic activity of CYP2C19 was also assessed. Sex is an important factor in activity of some cytochrome P450 enzymes. CYP3A4 is an example of cytochrome enzymes which has higher activity in women than men [2]. There were some controversies in the previous published reports for impact of sex on CYP2C19 activity. Ramsjö *et al*. indicated a sex difference in CYP2C19 activity between Korean subjects and not in Swedish volunteers using Omeprazole as a probe drug [27]. Tamminga *et al*. observed a sex related decreased CYP2C19 activity in women when used mephenytoin as a probe for evaluation of CYP2C19 activity, however the author declared this reduction was more obvious in those who used oral contraceptive [42]. In contrast, Hägg *et al*. did not see any sex differences in CYP2C19 activity after administration of mephenytoin in Norwegian population [43]. By considering these reports one may conclude that sex dependency of CYP2C19 activity is influenced by environmental and epigenetic factors like diet and ethnic differences. The other possibility can be attributed to some new genetic mutation in some populations which has not been studied well. The result of this study represents no effect of sex on CYP2C19 activity which is in line with what is reported in Swedish and Norwegian population.

The frequency of PM and URM in Iranian population in this study was about 2.2% and 5.5% which is close to the study by Zand *et al.* [44] and other Caucasian population. In previous report by Akhlaghi *et al*. in Iranian patients with coronary artery disease the genotype frequency of CYP2C19*2*2 was reported 4.7% which is quite different from our results [45]. This can be due to difference in studied population (healthy volunteer’s vs specific patients). The genotype frequency of CYP2C19*17*17 (URM) was 4% in Swedish and 3% in Ethiopian [17], 5.1% in Danish [19], 3.18% in Greece [39], 7% in Saudi Arabian [46] 1.2% in Indian [16] and 0% in Japanese, Korean and Thai population [20,27,47]. Accordingly the genotype frequency of CYP2C19*2*2 (PM) is 2.2% in Danish [19], 2.1% in Greece [39], 0.4% in Saudi Arabian [46], 18.4% in Indian [16] and 18% in Japanese people [20].

To the best of authors’ knowledge, this is the first study evaluating CYP2C19 genotype and phenotype in Iranian population in relation to new variant allele (CYP2C19*17). In the previous report, Zendehdel *et al.* investigated impact of CYP2C19 on therapeutic efficacy of omeprazole in Iranian patients with erosive reflux esophagitis; patients were genotyped only for CYP2C19*2 and CYP2C19*3, Individuals with HetEM genotype had better response to treatment with omeprazole than EM (95% vs 43% successful treatment response respectively) [48]. The high frequency of CYP2C19*17 allele (21.6%) detected in this study maybe one justification for 50% resistance rate in the EM group in Iranian patients in previous report. However impact of this variant allele (CYP2C19*17) on the efficacy of PPIs like omeprazole shall be evaluated in controlled clinical trials.

In this study the antimode of 0.8 was calculated for Iranian population. Different antimodes for HI of omeprazole have been reported in different ethnics groups: 14.4 in Indian [16], 7.0 in Koreans [25] and Thai population [47], 0.63 in Colombians [26], and 3.98 in West Mexicans [49]. The calculated antimode for Iranian population is similar to Colombian population, indicating comparable CYP2C19 activity in Iranians and Colombians and faster enzyme activity than Asian people.

A complete genotype phenotype correlation was observed in this study. However it should be noted that enzyme activity and therefore metabolic ratio may vary during some disease condition which may result in discrepancy in genotype-phenotype relationship of specific enzyme. Kimura *et al.* [50] indicated discordance of genotype-phenotype relationship of omeprazole in 14.5% of EM patients who had peptic ulcer disease. However this discrepancy was not observed in healthy individuals. Reduced hepatic enzyme activity as a result of old age or liver disease was reported as an explanation for such finding. Long term treatment with omeprazole which has auto inhibition effect was the other possibility. Williams *et al.* [51] also did not see genotype-phenotype relationship of 2C19 using omeprazole in patients with advanced cancer. The authors concluded that increased level of some signaling molecules like interleukin (IL) and tumor necrosis factors (TNFα) may result in down-regulation of metabolizing enzymes [51,52]. So it should be considered that factors like age, disease state, and concomitant medication may have pronounced effect on enzyme activity. Although omeprazole hydroxylation index has been used as an indicator of CYP2C19 activity, it should be considered that hydroxyomeprazole which is formed by CYP2C19 is further metabolized by CYP3A4 to hydroxyomeprazole sulfone [53] which in turn may indirectly affect the hydroxylation index of omeprazole. Therefore the high concentration of CYP3A4 in liver microsomes of some human can explain the deviation from CYP2C19 genotype and also the sex dependent enzyme activity observed in some ethnic groups [54].

The prevalence of CYP2C19*2*17 in this study was only 6% which is a limitation of this study. Future studies to investigate impact of CYP2C19*2*17 genotype on CYP2C19 enzyme activity in larger groups specially by using drugs with narrow therapeutic window is suggested.

In conclusion, the result of this study shows that CYP2C19*2*17 has an intermediate metabolic activity which maybe important for drug dose adjustment regimens for treatment, specially in those having narrow therapeutic indices like clopidogrel. Additionally no effect of sex on CYP2C19 activity was observed in this study. Regarding the high frequency of CYP2C19*17 in Iranian population, the importance of this new variant allele in metabolism of CYP2C19 substrates shall be considered.

## Abbreviations

CYP2C19: Cytochrome P450 2C19
PCR-RFLP: Polymerase chain reaction-restriction fragment length polymorphism
HPLC: High performance liquid chromatography
HI: Hydxroxylation index
SNP: Single nucleotide polymorphism
URM: Ultra-rapid metabolizers
EM: Extensive metabolizers
IM: Intermediate metabolizers
PM: Poor metabolizers
OMP: Omeprazole
OH-OMP: Hydroxyomepazole
